# Research on quality evaluation of innovation and entrepreneurship education for college students based on random forest algorithm and logistic regression model

**DOI:** 10.7717/peerj-cs.1329

**Published:** 2023-04-17

**Authors:** Qianqian Lu, Yongxiang Chai, Lihui Ren, Pengyu Ren, Junhui Zhou, Chunlei Lin

**Affiliations:** 1 Administrative Office, Zhejiang Guangsha Vocational and Technical University of Construction, Dongyang, China; 2 School of Information, Zhejiang Guangsha Vocational and Technical University of Construction, Dongyang, China; 3 Department of Public Physical Education, Zhejiang Guangsha Vocational and Technical University of Construction, Dongyang, China; 4 Department of Education and Engineering, Zhejiang Guangsha Vocational and Technical University of Construction, Dongyang, China

**Keywords:** Innovation, Entrepreneurship, Random forest, Logistic regression, Entrepreneurial education, Curriculum development

## Abstract

The quality evaluation of innovation and entrepreneurship (I&E) in the education sector is achieving worldwide attention as empowering nations with high quality talents is quintessential for economic progress. China, a pioneer in the world market in almost all sectors have transformed its educational policies and incorporated entrepreneurial skills as a part of their education models to further catalyst the country’s economic progress. This research focuses on building a novel hybrid Machine Learning (ML) model by integrating two powerful algorithms namely Random Forest (RF) and Logistic Regression (LR) to assess the intensity of the I&E in education from the data acquired from 25 leading Higher Educational Institution’s (HEI) in different provinces. The major contributions to the work are, (1) construction of quality index for each topic of interest using individual RF, (2) ranking the indicators based on the quality index to assess the strength and weaknesses, (3) and finally use the LR algorithm study the quality of each indicator. The efficacy of the proposed hybrid model is validated using the benchmark classification metrics to assess its learning and prediction performance in evaluating the quality of I&E education. The result of the research portrays that the universities have now started to integrate entrepreneurship skills as a part of the curriculum, which is evident from the better ranking of the topic curriculum development which is followed by the enrichment of skills. This comprehensive research will help the institutions to identify the potential areas of growth to boost the economic development and improve the skill set necessary for I&E education among college students.

## Introduction

Innovation and entrepreneurship (I&E) is an important driver to accelerate the economic growth, population integration, social mobility, cultural formation and financial immunity of any country (Wang, 2022). They form the key elements of digital age. More precisely, the culture of I&E supports the country to combat the economic instability and shock caused due to unprecedented conditions like the pandemic by transforming the genre of education through innovations which greatly relieves the employment pressure. China has emerged as a forerunner in cultivating and incubating the entrepreneurship qualities of the students by reforming the country’s educational policy. In spite of the efforts took by the state and Higher Educational Institutions (HEIs), the development of I&E is still in its stage of infancy. According to the Chinese College Students Entrepreneurship Report published in 2021, nearly 96.1% of the students from HEIs have good entrepreneurial ideas, out of which only 14% of the students are able to realise it. Globally, China has reached second position in I&E during 2021 from being eleventh in the year 2002 (Huajie et al., 2022). The increasing rise of I&E education in China is aligned towards building innovation-oriented country by promoting high-quality education which imbibes holistic knowledge about economics, science and technology, employ ability skills, analysis of job markets, and formalizing the employment structures. The Ministry of Education has issued certain significant key points in April 2012 such as:

• I&E should be implemented in HEIs through personnel training
• Channelizing fundamental requirements on I&E in HEIs
• Developing world-class curriculum with a vision of I&E
• Training teachers
• Extending hands to students with good I&E skills.

The various stakeholders which are a dynamic attribute are listed in Table 1. The list is not exhaustive but throws light on few examples where the interest of stake holders deserves special mention.

Invigorating the I&E education will be the promising direction for educational reforms to envisage a holistic economic growth. Integrating I&E as a part of professional education has emanated as a new challenge to developing the curriculum. New policies, views and insights are quintessential to implement vocational knowledge; improve the quality of technical and digital talents to integrate innovation education and entrepreneurship education into professional education. Almost all the HEIs in China have initiated to promote I&E as a part of the education to eventually improve the quality of entrepreneurial abilities among students. Nevertheless, hurdles such as proper design of curriculum, training the mentors/ teachers and provision resources are to be addressed by the HEI’s due to the dynamically evolving entrepreneurship nature.

**Figure 1:**
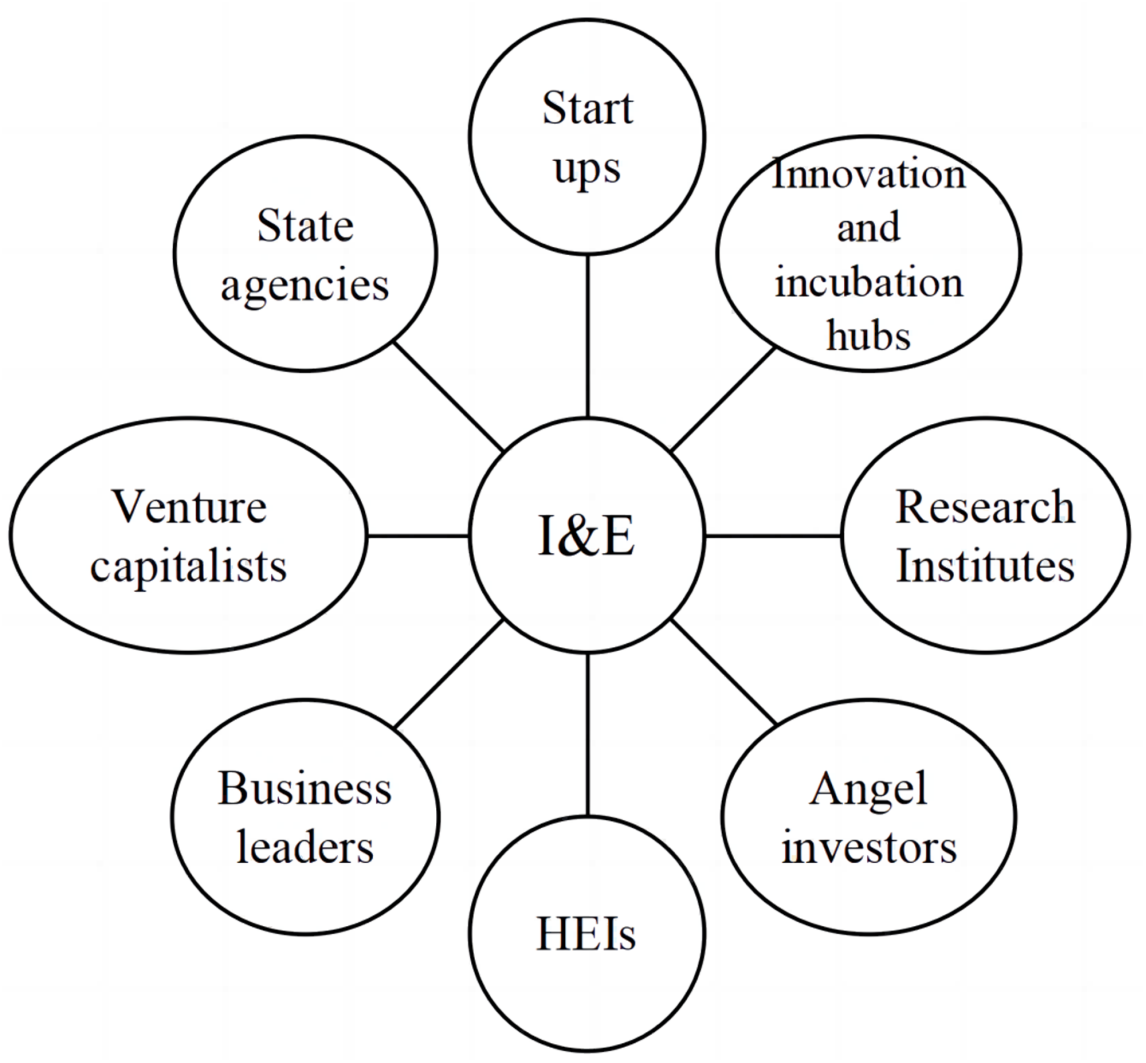
Stakeholders involved in I&E.

**Figure 2:**
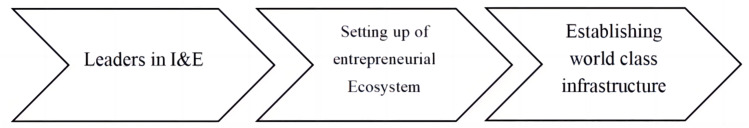
Milestones in achieving entrepreneurial excellence.

Promoting I&E in HEIs is a culmination of series of micro and macro managed activities that involves right amalgamation of various stakeholders as given in Fig. 1. Proper integration of the factors to develop I&E will eventually help the country to attain an excellency and become a leading pioneer in global entrepreneurship opportunities that can be realised through the activities mentioned in Fig. 2.

**Table 1:** Various stakeholders and their scope for business.

Stakeholder	Creates values for	Service	Scope
Well established business	Customers, client, employees and investors	Commercial products or services	Financial
Business entrepreneur	Customers, client, employees and investors	Novel Commercial products or services	Financial
Social entrepreneur	Society	Novel social services and products	Financial and social
State	Citizens	Welfare services	Cultural, financial and social
Student	Future employees	Adopting work life	Cultural, financial and social

**Figure 3:**
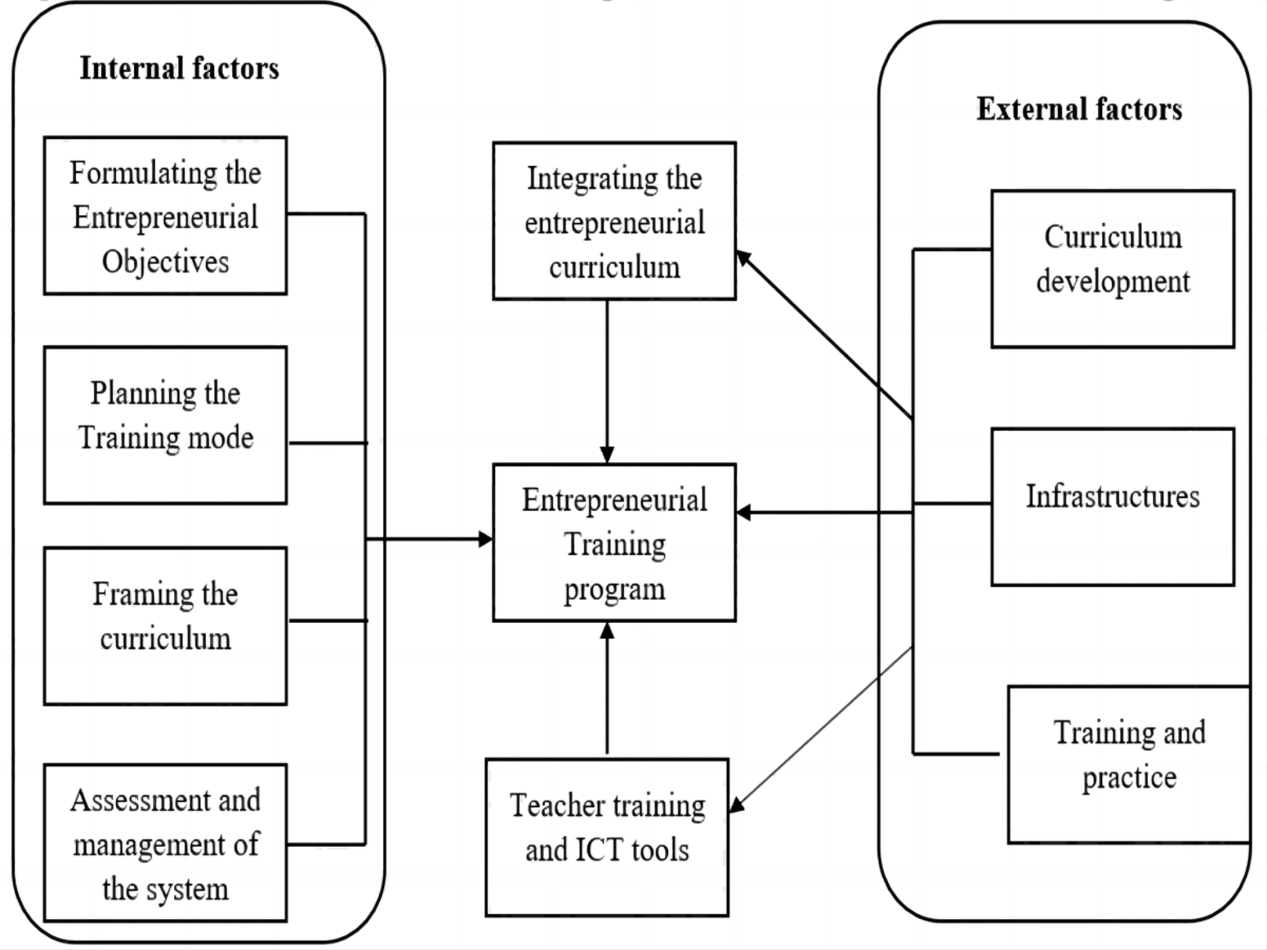
Internal and external factors involved in promotion I&E in education.

Quantifying and measuring the intensity of I&E in education is the greatest challenge faced by almost all HEIs (Wang & Liu, 2022). The Entrepreneurial Intention (EI) within the students is one of the best predictors to quantize the entrepreneurial behaviour (Lihua, 2022). In addition to this, the degree of correlation between the factors such as EI, feasibility and desirability must be high to incubate the entrepreneurial ecosystem in HEIs. In other words, EI imparts better entrepreneurship thus enabling or enriching the entrepreneurial activities (Liñán & Fayolle, 2015). Most importantly, entrepreneurship education (EE) is a tool to nurture entrepreneurship as it demands lifelong learning mandates (Boldureanu et al., 2020). China, has well-understood the significance of I&E and hence it integrates the EE into classrooms either as core or elective course for college students, helping college students better plan their time and future career development (Ratten & Usmanij, 2020). The ultimate objectives of all these innovative endeavors are to motivate the young students to start their own business ventures. The entrepreneurial teaching must incorporate business plans, develop entrepreneurship courses, and other related entrepreneurial talents training. Figure 3 (Karen, 2008) shows the factors that great influence the development of comprehensive entrepreneurial training in HEIs. A major transformation can happen by proper entrepreneurial training by shifting from the teacher central training to student central cultivation.

### Challenges confronted by I&E in Chinese HEIs

The evolution of I&E in China shows a very promising development. However, there are few points to be addressed to integrate I&E holistically as a part of the regular curriculum. Few salient points are mentioned here:

• Very limited number of courses and resources: US, a potential rival of China in the global market is offering much more entrepreneurial courses with excellent infrastructure (Yulian et al., 2014). This limits the opportunities of native students to develop their entrepreneurial talents.
• Lack of trained skillset: As it is evident that I&E is a comprehensive discipline that demands skilled manpower to train the students with all necessary traits. Unfortunately, the present trend indicates that there is scarcity of well-qualified teachers in this genre (Zhou, Li & Shahzad, 2021).
• Limited specializations: Some of the courses that are offered by pioneering universities in China centers on entrepreneurial management, business communication and practices, business finances. team and development training, entrepreneurial market analysis, corporate and contract law etc. These numbers are far lows than other developed countries.
• Single course structures: The conventional education coins around the traditional closed teaching model, which does not provision the inclusion of any new reforms. Integration of any new measures like embedding I&E through simulation, training or any practical experience is possible only in very few ventures (Guo, 2022).
• Lack of formal education model: In reality there is no formalized model that could guide the entrepreneurial teaching in the HEI’s. A wide gap can be sensed in integrating all the essential elements of teaching like curriculum development, teaching pedagogy, training and guidance schemes. Also, the implementation of the entrepreneurial education should always align to educational goals, program specific outcomes, and the assessments. EE in most of the Chinese universities are not adhering to these demands (He & Dong, 2022).
• Lack of long term goals and support mechanisms: Lack of long run goals in HEI’s and rushing behind immediate results ultimately leads to unsuccessful EE with no funding mechanism, hatch and policy safeguard which takes a great toll in the development of EE (Chen, 2022a; Chen, 2022).
• Insufficient funds: At present, funds in Chinese HEIs are utilized mainly to meet the need of entrepreneurship that are sourced by the government, private entrepreneurs, and very rarely from the funds allotted by schools (Han et al., 2018). These funds are highly insufficient.
• Lack of proper incubator space: Incubator space is the ground in which the I&E can be practised by the students. It can include laboratory space or office area where the student can practice business activities, perceive the real market with state of art technological innovation (Gong et al., 2020). Insufficient support system in China, has made these unrealistic due to various limitations like conditions, funds and lack of teachers etc.

The above-described issues are in the front line of the I&E in China. These challenges hinder the development of formalized curriculum as each of them are very versatile. Also, the development in I&E is happening in a very slow pace, as the issues can be combated in long run.In spite of all these bottlenecks the Chinese universities are gearing up towards developing I&E. Many researchers, scholars and experts are committed to model the innovative entrepreneurship traits in students with a more formalised research and analysis procedures, to evaluate the quality of I&E in HEI’s of China. A well-defined perfect I&E education quality assessment model will eventually help the community to cater the immediate needs of quality I&E.

### Educational data mining

**Figure 4:**
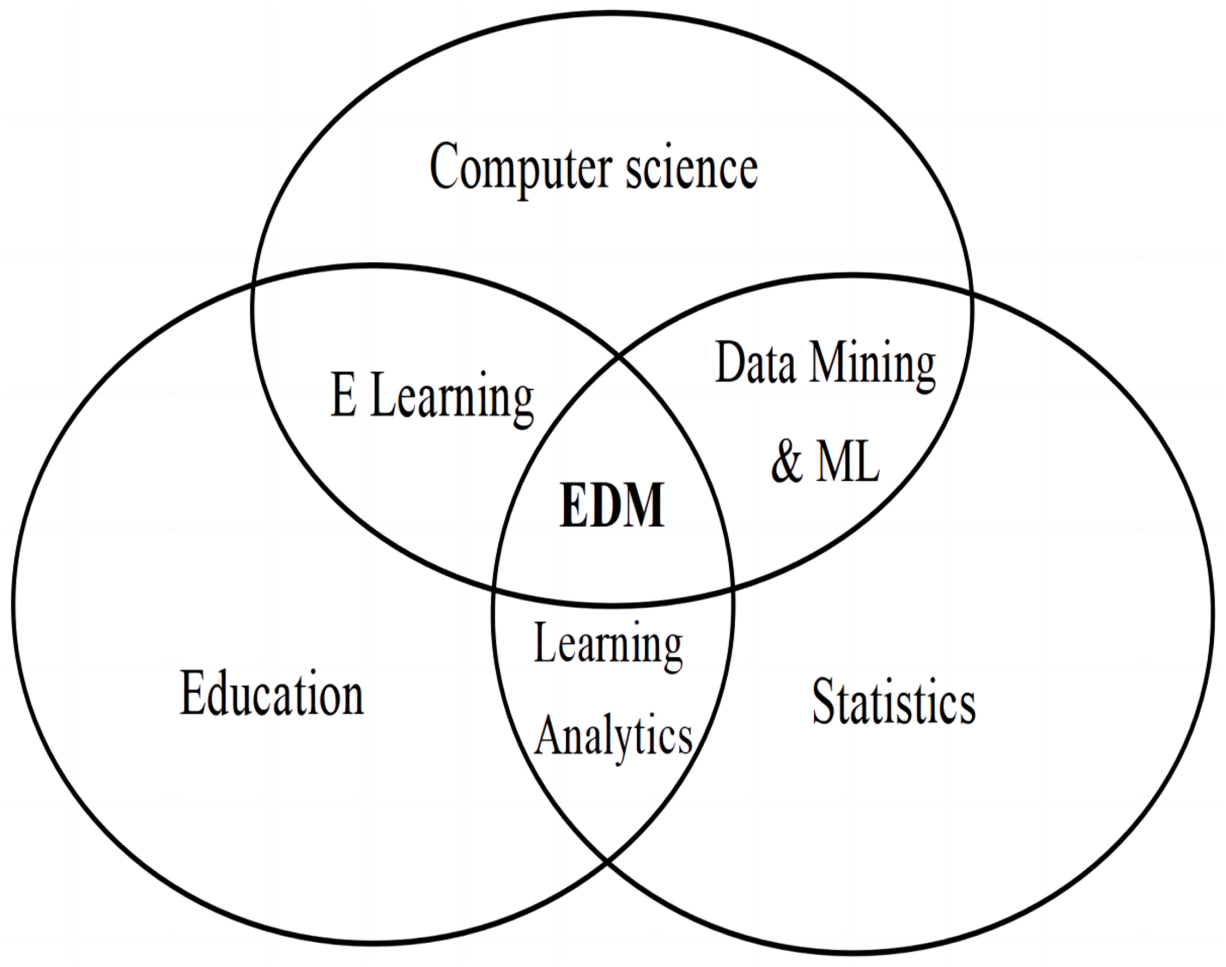
Educational data mining.

Modern developments in education have inspired the growth of Educational Data Mining (EDM). Multitude of research has enforced and identified new ventures and avenues for imparting technologically enhanced learning models and systems based on the societal and students’ needs. I&E, which is a new sector of education has leveraged the EDM to model more precise assessment systems. EDM is perceived as a holistic and comprehensive model that integrates valuable insights from multiple domains like Computer Science, Data Mining, E-Learning, data analytics, Machine Learning (ML) and statistics. The relationship among these domains is represented in Fig. 4 (Gordon & Helen, 2022). Among these, ML is a field that has witnessed rampant advancements which is further accelerated by learning analytics (Trakunphutthirak & Lee Vincent, 2022).

ML, a subset of Artificial Intelligence (AI) is the method of imparting power to the model or machine to access the data and learn the inherent patterns and trends by itself (Edwards Audrene, Kaplan & Jie, 2020). The field of ML progressed by programming the computers to optimize or fine tune a performance criterion through sample or example data (Alexandros & Alexandra, 2018). This model is generally perceived as the black box where the data enters the model in its beginning, and output is obtained out at another end. However, the processes that happen in the black box is quite complex. Few potential business implementations of ML in the field of education are:

• Predict student performance (Rastrollo-Guerrero, Gómez-Pulido & Durán-Domínguez, 2020), where a ML model would be deployed in predicting the performance of a student by learning the qualities of individuals.
• Assess students and grade them using ML model which creates automated adaptive assessments (Liu & Ardakani, 2022). This model will provide continuous feedback to the teachers about the student’s learning ability and their progress towards learning goals.
• ML models are used to predict the retention rates, which helps the institution to adapt strategies to help students to continue their education (Samer et al., 2022).
• Automatic evaluation by deploying handwriting recognition systems (Vij, Tayal & Jain, 2020).
• Quality assessment about the course from the formal and informal feedback and student qualities (Li, 2022).

Literature in the field witnesses the deployment of all types of ML algorithms namely supervised unsupervised and semi supervised algorithms. Mathematically, the supervised algorithms predict the output by learning the history of previous input and output. The model’s mapping will be Y =f(x) where Y is the output to be predicted based on the input variable/ variables (x). On the other hand, unsupervised learning algorithms tends to explore the relationships between the inputs and predict the output. The training data will not contain output component. The semi supervised learning is an amalgamation of the former methods, where only few data will contain outputs. A wide variety of ML algorithms like back propagation network, Linear regression, Logistic Regression (LR), Support Vector Machine (SVM), Decision trees, etc.

This work focuses on applying ML algorithms to analyse the quality of I&E education among the College Students. The biggest challenge faced by the educationalists and entrepreneurs is the lack of formal and tractable assessment method that learns the qualitative data to assess the quality of I&E education. The proposed research works attempts to analyse and explore the survey results, which is procured from nearly 25 HEI’s in China. The questionnaire majorly focused on assessing the quality on five significant domains namely Curriculum Design, Training and skill set enrichment, Resources, Talent acquisition by teachers and setting Entrepreneurial environment. The responses were scaled and fitted individually into RF algorithm is to construct a novel Quality Index (QI) that characterizes the eminence of the institutions. This QI is learnt by the RF algorithm by number of individual Classification and Regression based DTs with pruning. Ensemble of the results of the DTs will output the QI, a regressive value. This QI is then fitted into an LR model that finally evaluates the Total Quality Evaluation (TQE) based on the results of applying sigmoid function on the linear regression expression.The major research contributions of the proposed work are:

• A comprehensive analysis of the factors affecting the EE and the hurdles faced by the HEIs in incorporating EE as a part of the curriculum.
• Formulation of the assessment criteria with its relevant indicators
• Development and implementation of Random Forest (RF) and LR model for evaluating the quality of the I&E education
• Validating the models based on standard performance measures

The organisation of the work is as follows: Section 2 gives a brief outline of the important related works. Section 3 presents the information about data sourcing and research methodology. Section 4 portrays the results and the important implications. Conclusions and future research directions are given in Section 5.

## Related Works

Literature witnesses the presence of notable works in the field of assessing the performance of students, curriculum and other entrepreneurial elements offered by the HEIs. Some of the prominent works are briefed in this section.

Huang et al. (2019) developed a BackPropagation (BP) model with optimization to assess quality of I&E using an evaluation index system. The data was collected from major engineering colleges and HEIs to find that the innovative methods must be implemented to foster the I&E in HEI’s. Another notable work using back propagation network on quality assessment is done by Wang et al. (2022a) and Wang et al. (2022b). This empirical study is primarily focused on qualitative and quantitative assessment made on local University students. A fuzzy based fault tree analysis approach is deployed by Wang et al. (2022a) and Wang et al. (2022b) for assessing the reliability of I&E in classrooms. The efficacy of the model is validated using a comprehensive questionnaire. A holistic platform and core business processes for assessing the quality of I&E in HEI’s is proposed by Chen (2022a) and Chen (2022). The proposed system integrated many modules like skill diagnosis, project management skills, online teaching-learning, team building resource availability, testing and assessment evaluation, information and resource sharing etc. A BP model is used to verify the findings of the research.

A novel ML based system is proposed by Jiang & Li (2021) that considers the curriculum, teaching and training modes, reforms in practice and teaching in I&E. An intelligent strategy to assess the performance is designed using SVM, Extreme Learning Machines (ELM) and Radia Basis Function (RBF) that uses the facial expressions of the students. An improved collaborative filtering model that uses behavior graph and route of students to assess the performance of EE (Geng, 2022). This path is and the multidimensional behavior path is analysed for extracting the similarities between each dimension. A deep learning model that relies on human comprehensive development to assess the innovative quality in EE is designed by Zhang (2020). This model understands the learning status of students to assess the quality of I&E. A deep learning approach that is rooted on the understanding, creation and application of knowledge in I&E was proposed by Jing (2022). This model analyses the performance by monitoring the training, critical thinking, knowledge transfer, resource utilization, availability, flexible learning methods etc as the performance indicators. A deep learning model based on the empirical and theoretical research foe assessing the quality of I&E was proposed by Li & Zhao (2021). The learning algorithm models the experimental data to predict the quality.

Principle Component Analysis (PCA) and Linear Discriminant Analysis (LDA) was employed by Chen (2022a) and Chen (2022) to check the effectiveness of the I&E in modern education system. Entrepreneurship orientation among the individuals is studies and analysed using the predictive analytics approach using ridge, lasso, SVM, BP, and RF (Sabahi & Parast, 2020).

Graham & Bonner (2022) designed a decision tree approach on a large-scale dataset to monitor the I&E. The result indicates that entrepreneurial self-efficacy, networks and cultural perceptions are more dominant factors that determine the quality of education. The work by Qing & Zejun (2021) built ML based entrepreneurial policy analysis model to study the effect of entrepreneurial policies. The projection pursuit is optimized by applying genetic algorithm and results are validated using Entrepreneurial Activity index. RF is deployed to assess the level of entrepreneurial knowledge possessed by various sectors of students (Setiawan, 2021). Integrating the autonomy, critical thinking and automation in I&E using intelligent machines is described by (Knox, 2019).

The brief literature in the field of deploying I&E education among students in HEIs using ML, Deep Learning and other Artificial Intelligent techniques shows that there is still room for improvement by including more features that necessarily depicts the quality of the education mode.

## Data and Methods

### Data collection

The data was collected with the informed consent of the participants. Participants choose “Yes” or “No” in the questionnaire to agree or reject the questionnaire. Procedures associated with data collection were approved by the Zhejiang Guangsha Vocational and Technical University of Construction of Technology Committee.

The data for this study is procured from 25 colleges in China through questionnaires that will best describe the opinion of students about the I&E culture promoted in their institutions. Data collection was done randomly irrespective of gender in all fields of study like literature, language, Science and humanities, economics, law, management, arts, education, agriculture, Engineering, medicine etc. The students were asked to express their views in five scales as given in Table 2. The comprehensive questionnaire contains questions that assess the quality of I&E in various dimensions such as curriculum design, building entrepreneurial skills, resource availability, talent acquisition by teachers and entrepreneurial environment as given in Table 3.

**Table 2:** Five-point scale of the survey questionnaire.

S.No	Explanation	Scale
1	Strongly Disagree	1
2	Disagree	2
3	Neutral	3
4	Agree	4
5	Strongly Agree	5

**Table 3:** Cronbach reliability analysis and explanatory indicators for the study.

Topic	Subtopic	CITC	*α* coefficient of deleted items	Cronbach *α* coefficient	Variable
Curriculum Design	The resources, contents and materials of I&E are complete	0.707	0.904	0.913	X1
	The teaching method is unanimous	0.577	0.908		X2
	The training and practices of the I&E method is good.	0.518	0.910		X3
	The contents of the curriculum have good relevance to professional courses	0.585	0.908		X4
	The curriculum is globally competitive	0.575	0.908		X5
Training and skill set enrichment	Proper and relevant training is incorporated as a professional activity	0.623	0.907		X6
	Training is given to students by well qualified trainers	0.555	0.909		X7
Resources	Sufficient resources like smart rooms, work spaces, project development platforms, activity rooms etc are provided	0.628	0.906		X8
	Usage of Information and Communication Technology (ICT) tools	0.474	0.911		X9
	Financial policy and support to I&E activities	0.592	0.908		X10
Talent acquisition by teachers	Well qualified teachers	0.684	0.905		X11
	Development of I&E culture in teachers	0.599	0.907		X12
	Enough number of teachers	0.659	0.906		X13
	Availability of teachers in all disciplines	0.738	0.903		X14
	Experience and research capability of teachers	0.625	0.907		X15
Entrepreneurial environment	Constitution of entrepreneurial clubs	0.473	0.912		X16
	Conduction of seminars, guest lectures, symposiums, contests, workshops, conferences and outreach activities.	0.421	0.913		X17

As can be seen from the Table 3, the reliability coefficient value is 0.913, greater than 0.9, indicating that the reliability quality of the research data is high. For the “*α* coefficient of deleted items”, the reliability coefficient does not increase significantly after any item is deleted, indicating that the item should not be deleted. For “CITC value”, the CITC value of analysis items is all greater than 0.4, indicating that there is a good correlation between analysis items and a good level of reliability. In summary, the reliability coefficient of the research data is higher than 0.9, which indicates that the data reliability is of high quality and can be used for further analysis (Eisinga, Grotenhuis & Pelzer, 2013).

### Methods

The research deploys two important ML algorithms namely Random Forest (RF) and Logistic Regression (LR) for assessing the effectiveness of I&E in colleges using the data collected from the questionnaires under the five topics of interest.

#### Overview of the random forest algorithm

RF is a supervised algorithm that absorbs training samples and then input (X) into each sample to get the one output result (Y), through a mapping (f: X ⟶Y). Once the mapping is established by the model, predictions (Y’) for any unknown sample (X’) can be obtained. RF can be used for both classification and regression problems. The fundamental unit of RF is the Decision Tree (DT) and it works by integrating bagging with a random subspace. This combination helps the RF to improve its prediction accuracy.

**Figure 5:**
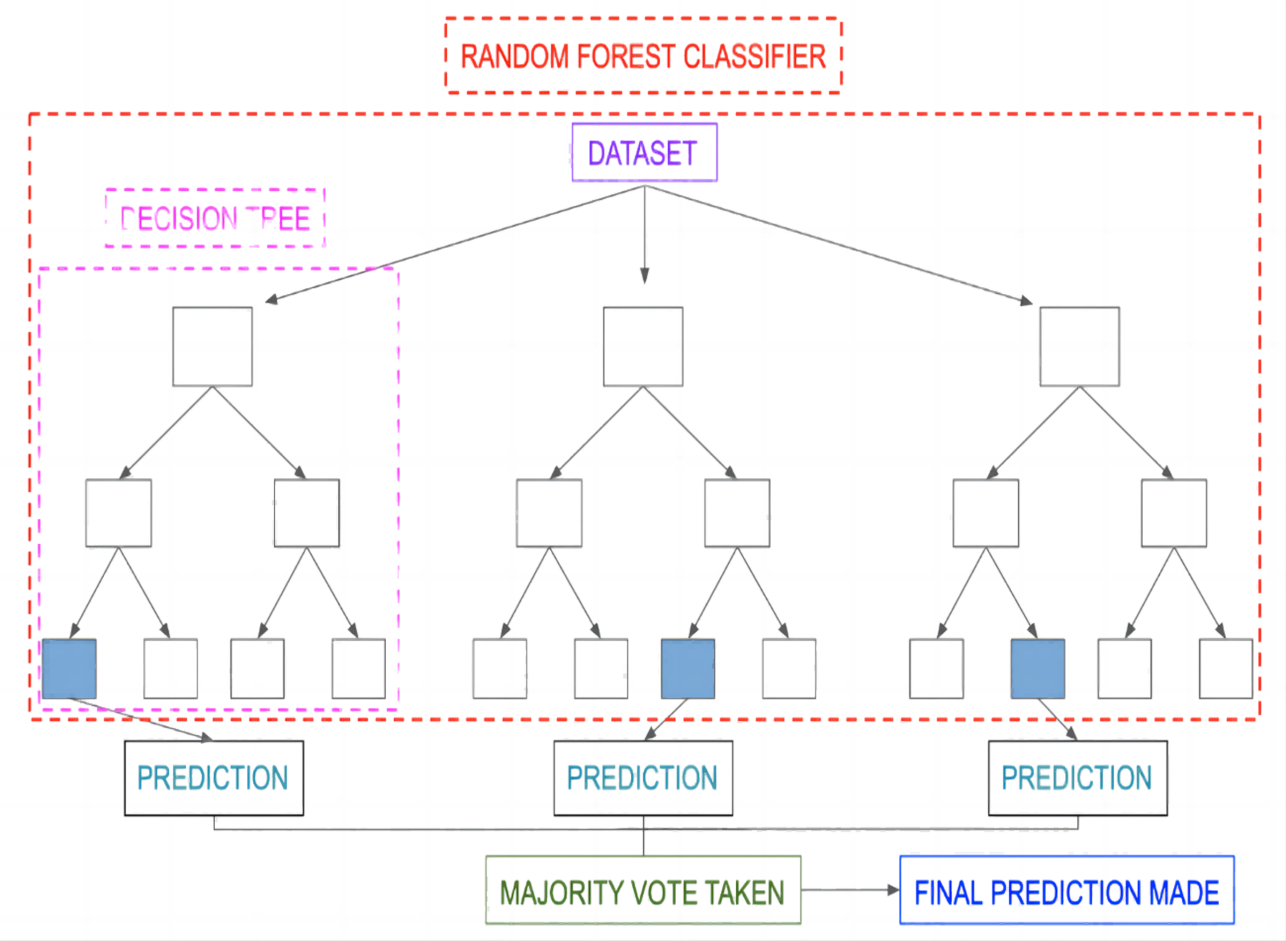
Outline of RF classifier.

The RF classifier integrates the decision of individual DTs d_1_(x), d_2_(x), …,d_n_(x) till sufficient accuracy is reached. This process is shown in Fig. 5 (Onesmus, 2020)*.* The results of the individual trees are combined through majority voting technique to give the result (Fawagreh, Gaber & Elyan, 2014). Two important phases in modelling RF are:

• Randomized training data: Bootstrapping method is employed in RF where the data samples are randomly picked by each DT.
• Randomized features: At each partitioning point of the individual DTs, the RF algorithm selects subset of features in random fashion without any replacement. K number of features will be selected, and then various indicators of the data will change under the K splits so as to choose the best split each time. This process will be continued till desired accuracy is reached.

The partitioning at each tree depends on two main metrics namely the entropy and information gain. The entropy of the sampled data T is found using Eq. (1).

(1) \begin{eqnarray*}\text{Entropy} \left( \mathrm{T} \right) =-\sum _{\mathrm{i}=1}^{\mathrm{n}}{\mathrm{P}}_{\mathrm{ i}}\log \nolimits 2~{\mathrm{P}}_{\mathrm{i}}.\end{eqnarray*}

The term P_i_ signifies the quantity of data samples belonging to a particular category i that contributes to the total samples. For every feature (A) the data will be sampled into k splits. The information entropy and gain will proceed according to Eqs. (2) and (3).

(2) \begin{eqnarray*}\text{Information Entropy}=-\sum _{\mathrm{j}=1}^{\mathrm{k}} \frac{ \left\vert {\mathrm{T}}_{\mathrm{j}} \right\vert }{ \left\vert \mathrm{T} \right\vert } \text{Entropy}(\mathrm{T})\end{eqnarray*}

(3) \begin{eqnarray*}\text{Information gain}=\text{Entropy(T)}-\text{Information Entropy}.\end{eqnarray*}

The variance or Mean Square Error is a measure that quantifies the best fit of the RF tree. It estimates the deviation of the estimated value from its actual value (Meyners & Hasted, 2022) and its expression is given in Eq. (4).

(4) \begin{eqnarray*}\mathrm{MSE}= \frac{1}{n} \sum _{i=1}^{n}({y}_{i-}\mu )^{2}.\end{eqnarray*}

The term y_i_ is the label of the n number of instances used for the construction of trees. The value *μ* represents the mean of the y_i_ for all the n instances considered in the study.

#### Overview of logistic regression

The logit or LR is a statistical model used for classification problems which estimates the likelihood of an event based on the independent variables (Niu, 2020). The result of LR is probability bounded within [0,1] and the transformation is obtained by the odds: ratio of success probability to the failure probability. Equation (5) depicts the Logit function.

(5) \begin{eqnarray*}\mathrm{Log} \left[ \frac{\mathrm{Y }}{(1-\mathrm{Y })} \right] ={\mathrm{b}}_{0}+{\mathrm{b}}_{1}{\mathrm{X}}_{1}+\ldots +{\mathrm{b}}_{\mathrm{n}}{\mathrm{X}}_{\mathrm{n}}.\end{eqnarray*}

The response variable (Y) is measured as the sigmoid function that estimates the values between [0-1]. The beta parameters are coefficients learned by the model using maximum likelihood estimation and are multiplied with the indicator or explanatory variables (X). The algorithm is run iteratively to find the best estimate. After obtaining the optimal coefficient the conditional probabilities for each record is determined and logged. The aggregation of this logged value will be the prediction or the response variable using the method of Goodness of fit.

#### Proposed model to assess the quality of Innovation and Entrepreneurship in college students

**Figure 6:**
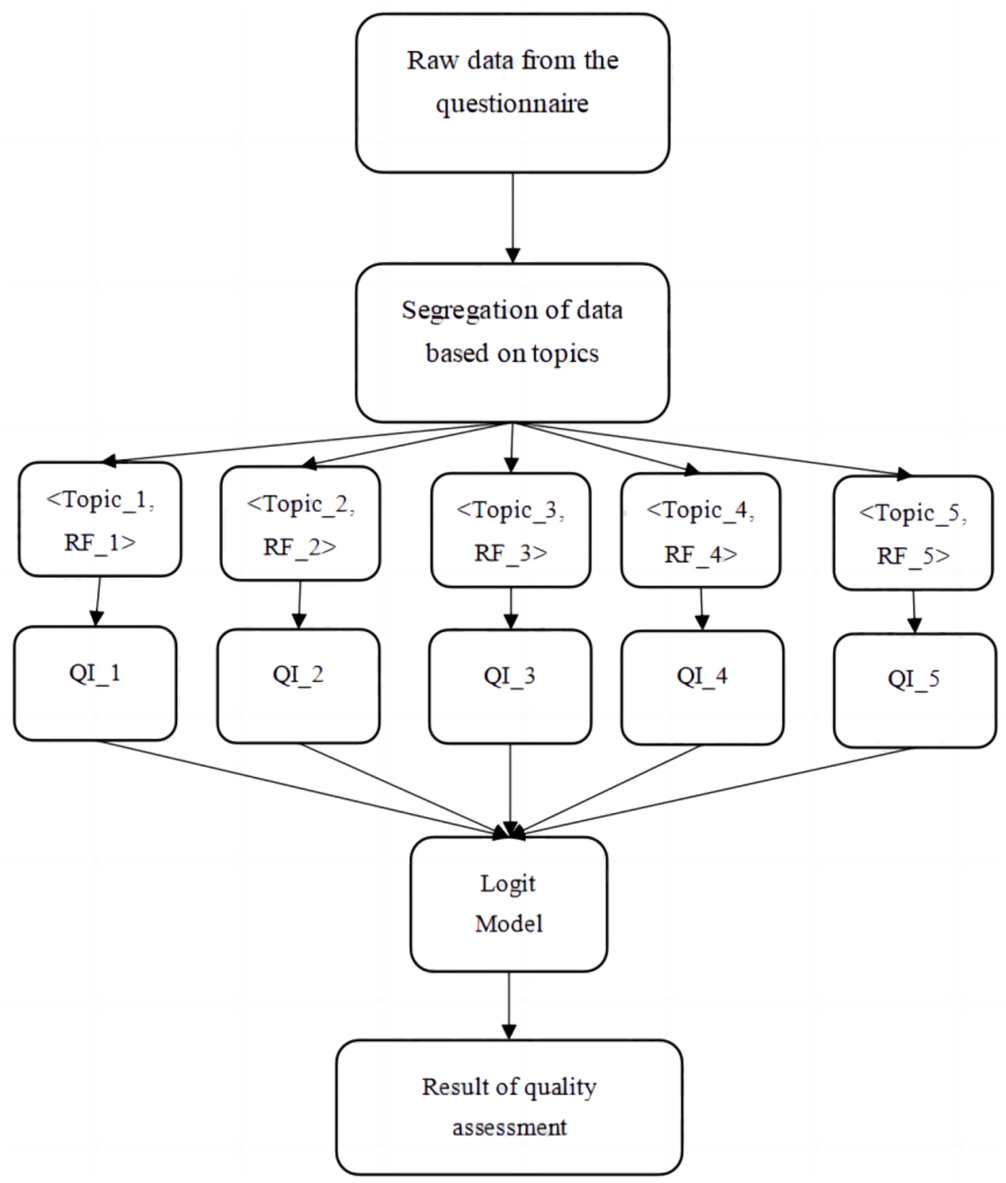
Proposed model to evaluate the quality of Innovation and Entrepreneurship using Random Forest and Logit model.

The research on evaluating the quality of I&E education based on the questionnaire collected from college students is done based on the model given in Fig. 6. This model integrated two ML algorithms namely RF and LR as discussed earlier. This hybrid model analysis the explanatory variables under five different genres namely Curriculum Design, Training and skill set enrichment, Resources, Talent acquisition by teachers and Entrepreneurial environment. Each of the topics is individually fit using RF algorithm to predict the Quality Index (QI). Each of the QI from QI_1 to QI_5 represents the quantitative value of the topic. The RF algorithm is an ensemble model that integrates individual decision trees till sufficient GI, Information gain and entropy is reached. This index is the quantitative metric that gauges the performance of the I&E of the colleges. To complete the research the QIs are fit using the Logit model to give the classification of the that is the final outcome. Table 3 lists the explanatory variables considered for the research.

The estimation of individual QIs are given in Eqs. (6) to (10).

(6) \begin{eqnarray*}QI\text{_}1=\text{Mean}(f\text{_}IDT(X1,X2,X3,X4,X5))\end{eqnarray*}

(7) \begin{eqnarray*}QI\text{_}2=\text{Mean}(f\text{_}IDT(X6,X7))\end{eqnarray*}

(8) \begin{eqnarray*}QI\text{_}3=\text{Mean}(f\text{_}IDT(X8,X9,X10))\end{eqnarray*}

(9) \begin{eqnarray*}QI\text{_}4=\text{Mean}(f\text{_}IDT(X11,X12,X13,X14,X15))\end{eqnarray*}

(10) \begin{eqnarray*}QI\text{_}5=\text{Mean}(f\text{_}IST(X16,X17)).\end{eqnarray*}

The proposed method uses predictive RF technique that averages the estimations of Individual Decision Trees (IDT) that are constructed in the implementation. Thus, it is evident from the Eqs. (6)–(10) that each RF explores the data space of the questionnaire independent of each other, to estimate the QI. Internally, the individual RFs namely RF_1, RF_2, RF_3, RF_4 and RF_5 were constructed using the information and entropy gain as discussed in the previous section. Constructing the trees based on these metrics will help the trees to assign higher adaptive weights to the instances or variables that holds greater significance. The models will be trained iteratively till better MSE is achieving, which is a direct implication of the error component between actual and predicted value.

The proposed novel hybrid model finally deploys LR or logit model that combines the statistically powerful linear regression and squeezes the estimated Y value into two classes. This result will be the comprehensive and holistic TQE that is the quality assessment outcome using the explanatory variables considered in this study. The estimation of TQE is given in Eqs. (11) and (12).

(11) \begin{eqnarray*}\mathrm{Y }={\mathrm{b}}_{0}+{\mathrm{b}}_{1}{\mathrm{QI}}_{1}+{\mathrm{b}}_{2}{\mathrm{QI}}_{2}+{\mathrm{b}}_{3}{\mathrm{QI}}_{3}+{\mathrm{b}}_{4}{\mathrm{QI}}_{4}+{\mathrm{b}}_{5}{\mathrm{QI}}_{5}\end{eqnarray*}

(12) \begin{eqnarray*}\mathrm{TQE}= \frac{1}{1+{\mathrm{e}}^{-\mathrm{Y }}} .\end{eqnarray*}

The coefficients b_0_, b_1_, b_2_, b_3_, b_4_ and b_5_ are computed adaptively by the login algorithm by training over the presented instances of the study. The proposed novel hybrid model is is motivated to achieve the following three major goals:

1. Prediction of the quality of I&E in education using the hybrid model constructed using RF and LR.
2. Assessment of individual topic of interest by constructing the QI using the RF algorithm.
3. Ranking the HEI’s performance in various topics as mentioned in Table 3.

## Results

The training and test data is taken in the ratio of 80:20. In the pre-processing step the data is segregated into five topics and each of them is fiting individually using five RFs. Table 4 presents the ranking of the topics based on the predicted indicator (QI_1 to QI_5). The individual estimator in each RF algorithm are taken as 10, which is a hyper parameter that is learnt by the proposed ML model after series of iterations. The label for the training data is computed based on the weighted scales as mentioned in Table 2 scaled between 0-1.

The results indicate that the HEIs are more focussed on talent and skill enrichment as most of the students who answered the questionnaire gave better rating to the training programs which is due to the enforcement of vigorous reforms in educational policies in China (Xiong, Yang & Shen, 2022). However, the country still has room for developing the curriculum design and constructing a good entrepreneurial ecosystem for the young students. Also, the performance of the hybrid model in assessing the quality of I&E in education is validated using the following classification metrics: accuracy, precision, recall, F1-score and support (Mehbodniya et al., 2022).

**Figure 7:**
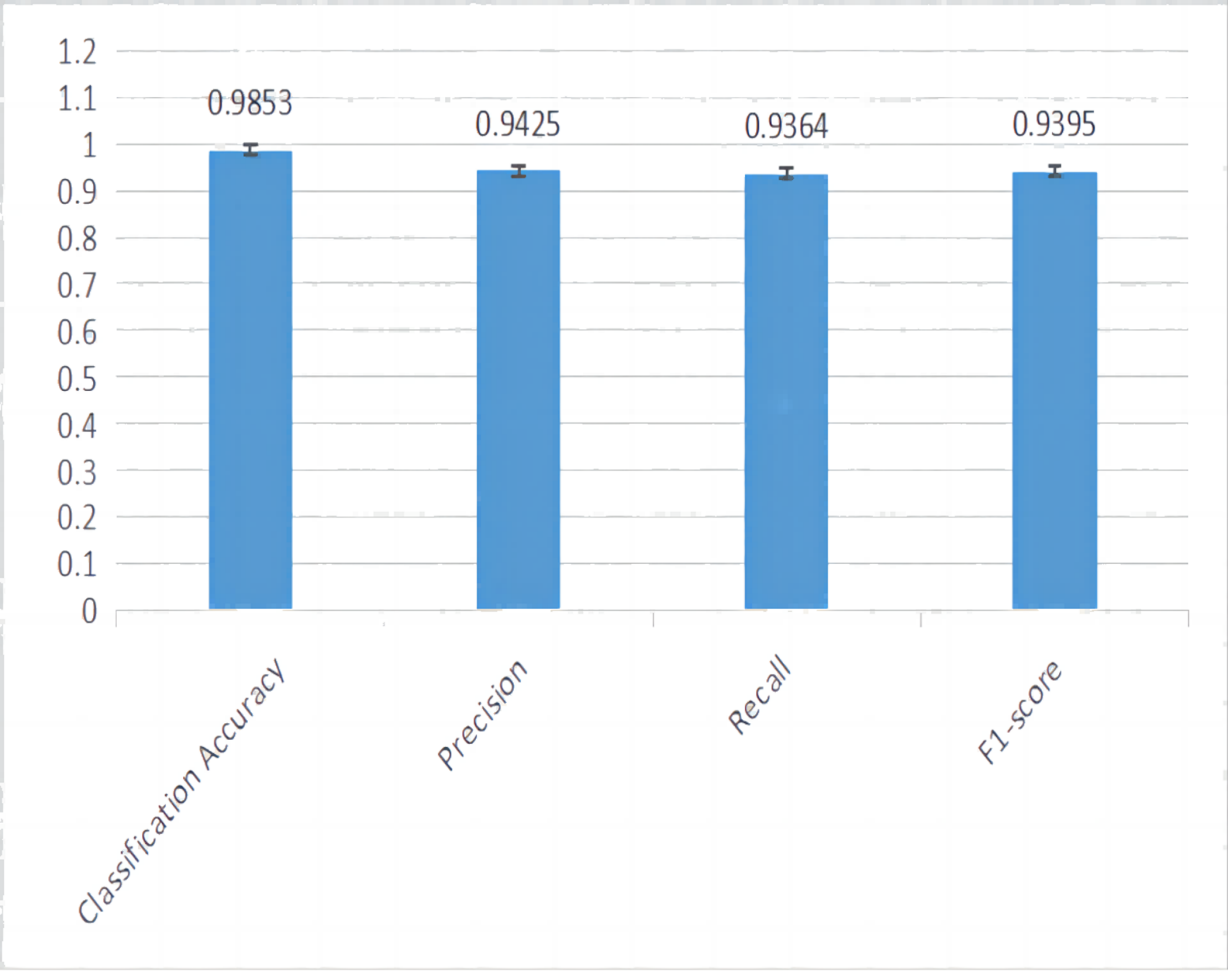
Graphical representation of the performance of proposed model.

The complete metrics of the proposed hybrid model are presented in Table 5. The hybrid model have predicted the quality with accuracy of 98.53% which is a promising value. The model also has very low misclassification rate that are witnessed through the high precision, recall and F1-Score values. The graphical representation of the efficacy of the model in evaluating the quality is given as Fig. 7.

**Table 4:** Ranking based on RF.

Rank	Topic	QI
1	Training and skill set enrichment	0.65
2	Resources	0.59
3	Talent acquisition by teachers	0.54
4	Curriculum Design	0.43
5	Entrepreneurial environment	0.23

**Table 5:** Summary of classification metrics.

S.No	Metric	Value
1.	Classification Accuracy	98.53%
2.	Precision	94.25%
3.	Recall	93.64%
4.	F1-score	93.95%

Table 6 shows the comparative performance analysis of the proposed work with other state of art techniques like ANN, SVM and DTs.It can be seen that SVM, ANN and individual DTs gives much lower accuracy when compared to the proposed work. This work trains individual DT on each topic and later combines the results of the trees to train the logit model. In addition to classification accuracy, the proposed work also assess the model’s performance on the misclassification rate that is ,assured through F1-score. The model has better F1-score of 93.95% followed by ANN model.

**Table 6:** Performance comparison with other state of art techniques.

S.No	Method	Classification accuracy	F1-Score
1.	SVM	90.81%	88.46%
2.	ANN	93.86%	90.68%
3.	DT	91.86%	84.13%
4.	Proposed work	98.53%	93.95%

The above research infers some important research implications. Table 7 gives the assessment results of the 25 HEI’s that are considered in this study.Few of the significant findings are explained below:

**Table 7:** Quality evaluation results of the research using the proposed hybrid ML model.

HEI	Ranking of the topics from (1-5)					TQE
	QI_1	QI_2	QI_3	QI_4	QI_5	
1.	1	4	2	3	5	Yes
2.	2	1	4	5	3	Yes
3.	3	1	2	4	5	No
4.	2	1	5	3	4	No
5.	4	3	5	1	2	No
6.	5	2	3	4	1	No
7.	1	2	5	3	4	Yes
8.	4	5	1	2	4	Yes
9.	4	2	3	1	5	Yes
10.	3	2	1	4	5	No
11.	4	1	2	3	5	No
12.	1	4	5	3	2	Yes
13.	5	2	3	1	4	Yes
14.	3	1	2	5	4	No
15.	4	2	1	3	5	Yes
16.	3	2	5	1	4	Yes
17.	2	3	1	3	5	No
18.	4	1	5	3	2	Yes
19.	2	4	1	5	3	No
20.	1	2	2	3	5	Yes
21.	2	3	4	1	5	Yes
22.	5	1	3	4	2	Yes
23.	2	3	4	5	1	No
24.	4	3	1	5	2	Yes
25.	3	1	2	1	5	No

• The results reveal that the overall impact on entrepreneurial education in terms of I&E in the colleges can be improved by integrating the elements of I&E as a part of the curriculum design based on the learning abilities and teaching efficiency of the students and teachers respectively. Also, it is important to include I&E in almost all disciplines of education based on the absorption ability and the skill set of the students. Special emphasis must be given to students in higher years of education to decide on their future career inclination either to start own enterprise or getting employed in firms.
• Colleges and HEIs should invite notable alumni, employment workshops, entrepreneurs, innovation camps, idea camps and experts with professional experience to foster the entrepreneurial ecosystem. This will motivate the students to combine their experiences and skills to understand and contribute to the development of enterprises.
• Special incentives and awards can be felicitated by the HEI’s to students who excel in I&E skills. This will aspire the student’s community to participate in more competitions and bring out innovative technology transfer ideas.
• Proper training should be given to teachers in transforming the teaching pedagogy from teacher centric to student centric notion. This will increase the student involvement.
• Multi-disciplinary and cross platform projects should be fostered and encouraged.
• HEIs must pay more attention to build world class laboratories, workspaces, project platforms and business simulation centres so that the student community can experience the business feel.

## Conclusions

The research on quality evaluation of I&E elements in education among the college students is empirically analysed using the hybrid RF- LR model. The educational reforms in China have opened doors for many regional and international students for pursuing entrepreneurial education. The data for the study is collected through a questionnaire and the responses are rated. The proposed model, (1) constructs individual QIs using RFs for five topics considered in the study, (2) ranks the QIs to understand the strength and weaknesses of HEI’s, (3) and fits a Logit model that classifies the quality of the I&E. The results indicate that training and skillset enrichment has been developed better than other classes. However, significant focus must be given by the government and HEIs in designing the curriculum and fostering entrepreneurial ecosystem. Also, the effectiveness of the proposed hybrid model is validated using classification metrics namely classification accuracy, F1-score, precision and recall. The results showed that the model showed promising results in assessing the quality of I&E in educational field among the college students. This research is focusses on questionnaires developed based only on six topics, which can be further extended to understand the I&E capability of students of various disciplines and the factors considered can include the cognitive and meta cognitive traits. And the combination of fuzzy algorithm (Dirik, 2022; Karahan et al., 2022) may make the data analysis more accurate, which is also where this paper will continue to study in the future.

##  Supplemental Information

10.7717/peerj-cs.1329/supp-1 Supplemental Information 1 Quality evaluation of innovation and entrepreneurship education in colleges and universities

## Additional Information and Declarations

### Data Availability

The following information was supplied regarding data availability: The raw data is available in the Supplemental Files.
