# Supplementary material for: Research on quality evaluation of innovation and entrepreneurship education for college students based on random forest algorithm and logistic regression model
Source: PeerJ Comput Sci. 2023 Apr 17;9:e1329. doi: 10.7717/peerj-cs.1329 (PMC10280670; doi:10.7717/peerj-cs.1329)
Supplement: Supplemental Information 1 [file peerj-cs-09-1329-s001.zip › Supplementary_file/code.docx]

import pandas as pd

import numpy as np

df = pd.read_excel('Quality evaluation of innovation and entrepreneurship education in colleges and universities.xlsx')

df.head(5)

df=df.drop(columns=['Serial number'])

df.head(5)

replace_dict={'Strongly Disagree':1,'Disagree':2,'Neutral':3,'Agree':4,'Strongly Agree':5}

df=df.replace({'3、Curriculum Design—The resources, contents and materials of I&E are complete':replace_dict,'3、The teaching method is unanimous':replace_dict,'3、The training and practices of the I&E method is good':replace_dict,'3、The contents of the curriculum have good relevance to professional courses':replace_dict,'3、The curriculum is globally competitive':replace_dict,

'4、Training and skill set enrichment—Proper and relevant training is incorporated as a professional activity':replace_dict,'4、Training is given to students by well qualified trainers':replace_dict,'5、Resources—Sufficient resources like smart rooms, workspaces, project development platforms, activity rooms etc are provided':replace_dict,'5、Usage of Information and Communication Technology (ICT) tools':replace_dict,'5、Financial policy and support to I&E activities':replace_dict,

'6、Talent acquisition by teachers—Well qualified teachers':replace_dict,'6、Development of I&E culture in teachers ':replace_dict,'6、Enough number of teachers ':replace_dict,'6、Availability of teachers in all disciplines':replace_dict,'6、Experience and research capability of teachers ':replace_dict,'7、Entrepreneurial environment—Constitution of entrepreneurial clubs ':replace_dict,'7、Conduction of seminars, guest lectures, symposiums, contests, workshops, conferences and outreach activities.':replace_dict})

df.head(5)

df_Q1=df.iloc[:,0:5]

Q1=df_Q1.sum(axis=1)/15

df_Q1

df_Q2=df.iloc[:,5:7]

Q2=df_Q2.sum(axis=1)/15

df_Q2

df_Q3=df.iloc[:,7:10]

Q3=df_Q3.sum(axis=1)/15

df_Q3

df_Q4=df.iloc[:,10:15]

Q4=df_Q4.sum(axis=1)/15

df_Q4

df_Q5=df.iloc[:,15:17]

Q5=df_Q5.sum(axis=1)/15

df_Q5

# Feature engineering: normalization

from sklearn.preprocessing import StandardScaler

standardScaler1 = StandardScaler()

standardScaler1.fit(df_Q1)

df_Q1 = standardScaler1.transform(df_Q1)

standardScaler2 = StandardScaler()

standardScaler2.fit(df_Q2)

df_Q2 = standardScaler2.transform(df_Q2)

standardScaler3 = StandardScaler()

standardScaler3.fit(df_Q3)

df_Q3 = standardScaler3.transform(df_Q3)

standardScaler4 = StandardScaler()

standardScaler4.fit(df_Q4)

df_Q4 = standardScaler4.transform(df_Q4)

standardScaler5 = StandardScaler()

standardScaler5.fit(df_Q5)

df_Q5 = standardScaler5.transform(df_Q5)

# Partitioning data sets

from sklearn.model_selection import train_test_split

train_X1,test_X1,train_y1,test_y1 = train_test_split(df_Q1,Q1,test_size=0.2,random_state=3)

train_X2,test_X2,train_y2,test_y2 = train_test_split(df_Q2,Q2,test_size=0.2,random_state=3)

train_X3,test_X3,train_y3,test_y3 = train_test_split(df_Q3,Q3,test_size=0.2,random_state=3)

train_X4,test_X4,train_y4,test_y4 = train_test_split(df_Q4,Q4,test_size=0.2,random_state=3)

train_X5,test_X5,train_y5,test_y5 = train_test_split(df_Q5,Q5,test_size=0.2,random_state=3)

# 【Random forest】

from sklearn.ensemble import RandomForestClassifier

from sklearn.metrics import classification_report

from sklearn.metrics import accuracy_score

rf1 = RandomForestClassifier(oob_score=True)

rf1.fit(train_X1,train_y1.astype('string'))

rf_pred_y1 = rf1.predict(test_X1)

print('Training set accuracy：

',rf1.score(train_X1,train_y1.astype('string')))

print('Test set accuracy：

',rf1.score(test_X1,test_y1.astype('string')))

print('Accuracy rate：

',accuracy_score(test_y1.astype('string'),rf_pred_y1))

print(classification_report(test_y1.astype('string'),rf_pred_y1))

rf2 = RandomForestClassifier(oob_score=True)

rf2.fit(train_X2,train_y2.astype('string'))

rf_pred_y2 = rf2.predict(test_X2)

print('Training set accuracy：

',rf2.score(train_X2,train_y2.astype('string')))

print('Test set accuracy：

',rf2.score(test_X2,test_y2.astype('string')))

print('Accuracy rate：

',accuracy_score(test_y2.astype('string'),rf_pred_y2))

print(classification_report(test_y2.astype('string'),rf_pred_y2))

rf3 = RandomForestClassifier(oob_score=True)

rf3.fit(train_X3,train_y3.astype('string'))

rf_pred_y3 = rf3.predict(test_X3)

print('Training set accuracy：

',rf3.score(train_X3,train_y3.astype('string')))

print('Test set accuracy：

',rf3.score(test_X3,test_y3.astype('string')))

print('Accuracy rate：

',accuracy_score(test_y3.astype('string'),rf_pred_y3))

print(classification_report(test_y3.astype('string'),rf_pred_y3))

rf4 = RandomForestClassifier(oob_score=True)

rf4.fit(train_X4,train_y4.astype('string'))

rf_pred_y4 = rf4.predict(test_X4)

print('Training set accuracy：

',rf4.score(train_X4,train_y4.astype('string')))

print('Test set accuracy：

',rf4.score(test_X4,test_y4.astype('string')))

print('Accuracy rate：

',accuracy_score(test_y4.astype('string'),rf_pred_y4))

print(classification_report(test_y4.astype('string'),rf_pred_y4))

rf5 = RandomForestClassifier(oob_score=True)

rf5.fit(train_X5,train_y5.astype('string'))

rf_pred_y5 = rf5.predict(test_X5)

print('Training set accuracy：

',rf5.score(train_X5,train_y5.astype('string')))

print('Test set accuracy：

',rf5.score(test_X5,test_y5.astype('string')))

print('Accuracy rate：

',accuracy_score(test_y5.astype('string'),rf_pred_y5))

print(classification_report(test_y5.astype('string'),rf_pred_y5))

df_new=pd.concat([Q1,Q2,Q3,Q4,Q5],axis=1)

df_new.rename(columns={0:'Q1',1:'Q2',2:'Q3',3:'Q4',4:'Q5'},inplace=True)

df_new

TQE=df_new.sum(axis=1)/5

TQE.loc[TQE>=0.6]=1

TQE.loc[TQE<0.6]=0

TQE

standardScaler = StandardScaler()

standardScaler.fit(df_new)

df_new = standardScaler.transform(df_new)

train_X,test_X,train_y,test_y = train_test_split(df_new,TQE,test_size=0.2,random_state=3)

# 【Logical regression model -- default parameters】

from sklearn.linear_model import LogisticRegression

# predictor

log_reg = LogisticRegression()

# Training model

log_reg.fit(train_X,train_y)

# Forecast data

log_pred_y = log_reg.predict(test_X)

# Evaluation model

train_score = log_reg.score(train_X,train_y)

test_score = log_reg.score(test_X,test_y)

print('Training set accuracy：',train_score)

print('Test set accuracy：',test_score)

print(classification_report(test_y,log_pred_y))
